# State-of-the-Art Molecular Plant Biology Research in Spain

**DOI:** 10.3390/ijms242316557

**Published:** 2023-11-21

**Authors:** Jesús V. Jorrin-Novo, Ricardo Aroca, María-Dolores Rey, Verónica Truniger, Pedro Martínez-Gómez

**Affiliations:** 1 Department of Biochemistry and Molecular Biology, University of Cordoba (UCO), Campus de Excelencia Internacional A3 (CeiA3), E-14014 Cordoba, Spain; bf1jonoj@uco.es (J.V.J.-N.); b52resam@uco.es (M.-D.R.); 2 Department of Soil and Plant Microbiology and Symbiotic Systems, EEZ-CSIC (Estación Experimental del Zaidin-Consejo Superior de Investigaciones Científicas), E-18100 Granada, Spain; ricardo.aroca@eez.csic.es; 3 Department of Stress Biology and Pathology, CEBAS-CSIC (Centro de Edafología y Biología Aplicada del Segura-Consejo Superior de Investigaciones Científicas), Campus Universitario Espinardo, E-30100 Murcia, Spain; truniger@cebas.csic.es; 4 Department of Plant Breeding, CEBAS-CSIC (Centro de Edafología y Biología Aplicada del Segura-Consejo Superior de Investigaciones Científicas), Campus Universitario Espinardo, E-30100 Murcia, Spain

## 1. Introduction

Molecular plant biology is the study of the molecular basis of plant life. It is particularly concerned with the processes by which the information encoded in the genome is translated to a specific phenotype, manifested as structures, processes, and behaviors. By using this approach, we strive to understand how the cellular building is constructed and how it works. The answer to this quandary is based on molecular interactions. Within this research, molecular biologists follow the central dogma of molecular biology, using in silico or wet approaches, including both classic and the most recent omics techniques that are integral to the path of systems biology. Different biological processes, from development and growth to interactions with biological, chemical, or physical surroundings, are nowadays approached by using molecular techniques in a number of different areas, including physiology, genetics, microbiology, cell biology, biochemistry, and molecular biology, among others. Even classic disciplines that are considered far from molecular biology use these approaches in cataloging living organisms. Apart from basic research that aims to generate knowledge, molecular biology techniques are the basis of biotechnology and offer solutions to the main problems and challenges that humans face, such as the provision of food, health care, environmental conservation, and the bioeconomy. With regard to the experimental system, the current projects and publications include model plants, crops, forest trees, and wild, non-domesticated species [1,2].

There are no major differences between the plant molecular biology research carried out in Spain and that in other countries, at least within industrialized regions. The only limitation and difference is posed by financial resources. This field has evolved with substantial progress in recent years around the world, and particularly in Spain, in order to decipher the molecular principles that underlie plant biology. The regulation of multiple pathways and regulatory targets for plant development, as well as adaptations to different stresses, have been elucidated during recent last years, enlightening the knowledge that we now hold about how plants cope with different environmental stimuli and how symbiosis with other organisms is used as a sustainable source of biostimulants in agriculture. The accumulated knowledge of plant molecular biology has also been enhanced due to improvements in molecular techniques and the biotechnology of plants for gene editing [1,3]. It is also important to mention the effort that has been undertaken in implementing the most recent techniques to orphan, recalcitrant, and non-domesticated species, as is the case with forest trees [4,5,6].

This review aims to provide a comprehensive overview of the recent advances in plant molecular science in Spain. These advances will be succinctly discussed in this review due to the increased interest within the plant scientific community in facing the significant challenges of the 21st century, in particular the need to increase the global food supply under the increasing threats of climate change.

## 2. Research Activity

Molecular plant biology research in Spain involves a great number of groups belonging to different research centers, mostly at the University and the Spanish Research Council known as the “Consejo Superior de Investigaciones Científicas” (CSIC) in Spanish, with institutes such as the Plant Biotechnology and Genomics Center (CBGP) in Madrid [7], the Institute of Molecular and Cellular Biology of Plants (IBMCP) in Valencia [8], the Centre for Research in Agricultural Genomics (CRAG) in Barcelona [9], the Institute of Plant Biochemistry and Photosynthesis (IBVF) at Sevilla [10], and Centre for Edaphology and Applied Biology of Segura (CEBAS) at Murcia [11] being the most reputed within plant research. Most of these groups meet periodically during the meeting of the Spanish Plant Molecular Group, with the last, number XVI, celebrated in Sevilla in September 2022, in which around 300 scientists participated, presenting more than 400 communications in the form of plenary lectures, oral communications, and posters [12]. 

This editorial, accompanying the SI “Plant Molecular Biology Research in Spain”, is a tremendous opportunity to recognize the work that is being carried out by the pioneers in the field, with a special mention to the groups led by Profs Francisco García Olmedo and Pilar Carbonero at Madrid, Pere Puigdomenech at Barcelona, Vicente Conejero at Valencia, and Manuel Losada and Enrique Cerda at Sevilla [12]. The earliest publications by Spanish groups appeared in the 1960s [13]. It is impossible to mention in this short review the many different contributions made by Spanish groups. In order to illustrate this, some figures are provided. Spain has one of the highest publication rates in plant sciences within the international community; sometimes, this appears to be a miracle, considering the financial support, which is considerably sparser than that of other EU or OCDE countries. Spanish groups have taken part in large consortiums, becoming involved in the Arabidopsis Genome Project in the 1990s [14], as well as contributing to the sequencing of important horticultural and fruit crops. Recently, for example, Rey et al. released the first draft of the genome of *Quercus ilex*, the most emblematic and representative species of the Spanish forest [15]. In addition, it is imperative to mention the leadership role played by Spanish groups within the plant proteomics arena [16].

**Figure 1:**
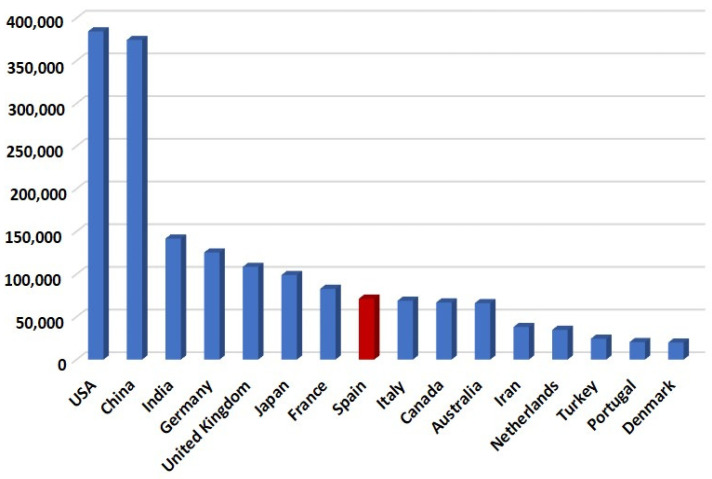
Number of documents related to molecular plant biology included in the Scopus database [17] from the largest-contributing countries in the world. In red the Spanish contributions.

In the 21st century, 73,263 documents have been published by Spanish teams in the field of plant molecular biology (search within all fields “Molecular Plant Biology” AND search within affiliation “Spain”) in the scientific database Scopus [17] (Figure 1), with 11,560 manuscripts (search all fields “Molecular Plant Biology” AND search address “Spain”) in the more restrictive Web of Science [18]. Within the global context, Spain occupies the eighth position after the USA (384,389 documents in Scopus [17]), China (374,307 documents), India (141,685), Germany (125,378), the United Kingdom (108,546), Japan (98,961), and France (82,809) in this regard (Figure 1). 

**Figure 2:**
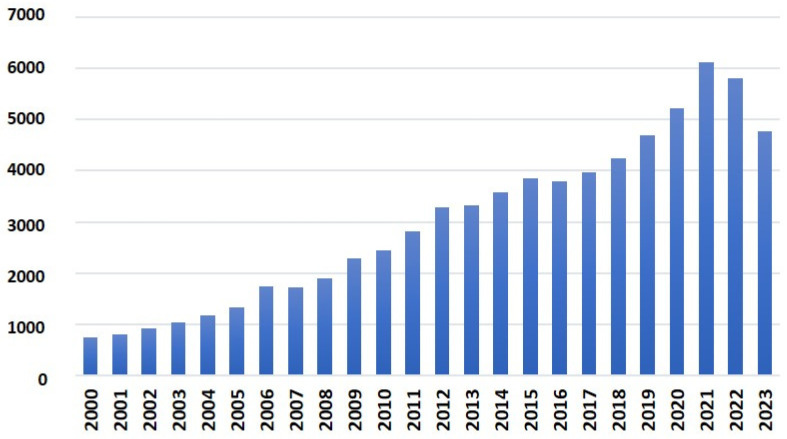
Evolution of the number of documents related to molecular plant biology in Spain included in the Scopus database [17] during the period 2000–2023.

The evolution of the publication of these documents is shown in Figure 2. Over the last 23 years, the number of publications has increased continuously, highlighting the strong position of Spain on the global stage.

**Figure 3:**
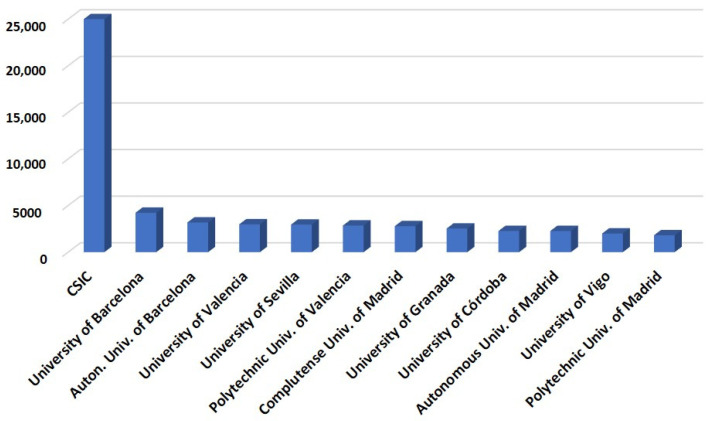
Number of documents related to molecular plant biology included in the Scopus database [12] from the most highly contributing Spanish universities and institutes.

On the other hand, regarding Spain’s main universities and institutes, the Spanish National Research Council (CSIC) which encompasses multiple research institutes) stands out, followed by the University of Barcelona, the Autonomous University of Barcelona, the Polytechnic University of Valencia, the University of Sevilla, the University of Valencia, the Complutense University of Madrid, the University of Granada, the University of Córdoba, and the Autonomous University of Madrid (Figure 3).

**Figure 4:**
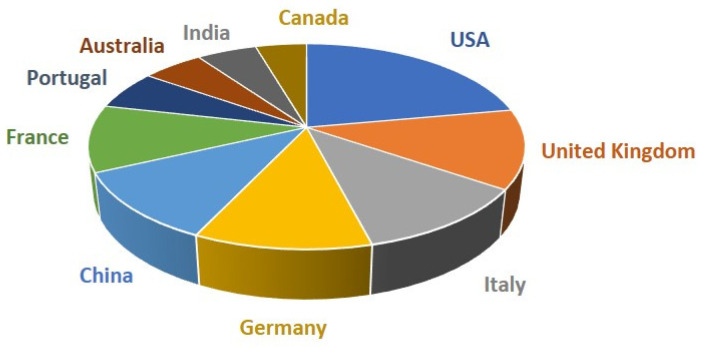
Percentage of documents from international collaborations of Spanish groups with the most prevalent countries included in the Scopus database [17].

Finally, regarding the international collaborations of Spanish groups, according to the Scopus database [17], around 70% of these studies have been performed with groups from other countries. Spain has collaborated the most with the USA (22% of documents from international collaborations of Spanish groups included in the Scopus database [17]) followed by the United Kingdom (13% of documents), Italy (12%), Germany (12%), China (11%), France (10%), Portugal (6%), Australia (5%), India (5%), and Canada (4%) (Figure 4). These data provide a solid understanding of the internationalization of studies within the field of molecular plant biology.

Additionally, Spanish research in plant molecular biology is attracting increasing attention within the enterprise community, as biotechnology research is extensively involved in product development for many agricultural, chemical, and pharmaceutical companies. In this context, a Spanish technological platform (BIOVEGEN) was recently created, bringing together private entities from the agri-food sector with interest in plant innovation, mainly plant biotechnology and plant molecular biology [19]. The main objectives of BIOVEGEN are to improve the competitiveness of the Spanish agri-food sector by incorporating new technologies based on plant molecular biology; to generate joint public–private R&D projects; to improve the transfer of knowledge and technology to the business sector; to contribute to the design of R&D strategies adapted to the agri-food sector; and to promote greater public and private investment in R&D. 

## 3. Research Areas

Meeting number XVI of the Spanish Plant Molecular Group, celebrated in Sevilla in September 2022, is the best example for illustrating the contribution of Spanish groups to the field of plant molecular biology. Specific sessions were devoted to systems biology, metabolism and signaling, development, plant–microbe interactions, abiotic stress, and biotechnology. Studies by Spanish groups have been focused on model experimental systems; Arabidopsis; unicellular algae such as Chlamydomonas; major crops; rice and other cereals; and, to a lesser extent, forest trees and other orphan and recalcitrant species, such as *Pinus* and *Quercus* [12]. In this context, we can summarize the most important research areas, including plant physiology and biochemistry, applied microbiology, genetics and heredity, cell and evolutionary biology, phytopathology, and environmental sciences.

### 3.1. Plant Physiology and Biochemistry

Plant biochemistry has been historically recognized as falling under the umbrella of plant physiology, whereas biochemistry was understood as mostly comprising that of microorganisms or animals. In the 1970s, only one session would be devoted solely to plants in biochemistry courses and textbooks: the study of photosynthesis. The first volume of the *Annual Review of Plant Physiology and Plant Molecular Biology* (formerly known as the *Annual Review of Plant Physiology*), appeared in 1988, having changed again to be known as the *Annual Review of Plant Biology* in 2004, and illustrates the evolution of the field. The close connection between plant physiology and biochemistry manifested through the establishment of journals such as *Plant Physiology and Biochemistry* in 1974. Nowadays, a high percentage of the papers published in top-ranking, historical journals in the plant science area, such as *New Phytologist*, the *Journal of Experimental Botany*, and *Plant Physiology*, have a clear biochemical and molecular biology focus, although it is true that it is difficult to establish clear limitations to specific areas as science becomes more and more transversal. 

It is important and a matter of justice to mention the Spanish pioneers in the field of Plant Biochemistry: Manuel Losada from Sevilla and Julio Lopez Gorge from Granada, among others. This classic focus is being continued by young Spanish scientists. The biochemistry, molecular biology, and biotechnology of important metabolites holding great health benefits, such as carotenoids, have been approached by different Spanish groups [20]. The group of Prof. A. Heredia at the University of Malaga has made important contributions to the chemical composition, molecular architecture, and biosynthesis of cutin, a topic that is under-considered within plant biology despite its importance in plant development and responses to stresses [21].

### 3.2. Applied Microbiology

Applied microbiology has also been an important area of study in Spain within the field of molecular plant biology. In recent years, several Spanish research groups have advanced our knowledge on how beneficial microorganisms in soil establish a molecular communication with host plants in order to form a favorable symbiosis. These signaling molecules include several flavonoid- and carotenoid-derived compounds, which can be used directly as biostimulants [22]. At the same time, progress in understanding how soil microorganisms induce abiotic stress tolerance in plants at the molecular level has been achieved, mostly focusing on drought and salinity tolerance [23]. Finally, there is a tight relationship between the root and soil microbiomes and their effects on plant health and development. In this field, several Spanish research groups have contributed to our understanding of how both microbiomes shape each other at the molecular level [24].

### 3.3. Genetics and Heredity

Through the application of molecular biology techniques, new plant varieties are being developed that are resistant to broad-spectrum herbicides, insects, viral pathogens, and extreme environmental conditions. The testing and analysis of genetically modified organisms (GMOs) using CRISPR technology has been efficiently carried out in Spanish laboratories [25,26,27]. In breeding programs, while the ability of breeders to generate large populations is unlimited, the management, phenotyping, genetic and inheritance study, and selection of these descendants are the main factors limiting the generation of new varieties that improve upon those that currently exist. Genomic (DNA) studies for the development of marker-assisted selection (MAS) strategies are particularly useful in the evaluation of different traits in the case of time-consuming phenotyping or in the case of tree species with long juvenile periods. More recently, proteomic (proteins and enzymes), transcriptomic (RNA), and epigenetic (DNA methylation and histone modifications) studies have been used in the mentioned genomic studies [25,28,29]. These are just a few examples of studies that have been authored or co-authored by Spanish scientists and groups. These are but a few of many; the limited size of this article makes it impossible to list them all. 

### 3.4. Cell and Evolutionary Biology

The production of new cells as a result of progression through the cell division cycle is a fundamental biological process for the perpetuation of both unicellular and multicellular organisms. In the case of plants, their developmental strategies and their largely sessile nature have imposed a series of evolutionary trends. In a Special Issue of the *International Journal of Molecular Science*s, “State-of-the-Art Molecular Plant Biology Research in Spain” [3], Crisanto Gutierrez [30] reviewed the current status of plant cell cycle studies as well as discussing early studies and the relevance of a multidisciplinary background as a source of innovative questions and answers. In addition to deepening our understanding of the machinery of the plant cell cycle, current studies focus on the intimate interaction of cell cycle components with almost every aspect of plant biology, with special emphasis being placed on Spanish works.

On the other hand, plant developmental biology in Spain is flourishing, with its future being highly dependent on appropriate funding conditions for its young scientists, the opening of new areas of research, the incorporation of technological breakthroughs into laboratories, and the undertaking of cooperative research by means of networking. In addition, the contributions of Spanish scientists to the advancement of plant developmental biology appears to be imbalanced towards reproductive biology, although relevant publications have also been reported on embryogenesis and seed development, tuberization, shoot branching, leaf development, signal transduction and hormone action, and the connection between growth and development [31].

### 3.5. Phytopathology

Research in plant molecular biology is also important for the study of pests and dis-eases in plants. Plant pathogenic microorganisms include fungi, viruses, bacteria, viroids, and oomycetes. These constitute an increasing serious threat to agriculture worldwide, especially due to climate change. Spain is one of the most important countries in agricultural production in Europe, often referred to as the orchard of Europe. Plant diseases are therefore extremely important in this regard, since they have the potential to lead to huge economic losses. There are a number of vital research groups interested in the field of plant pathology in Spain that focus on studying and combating plant diseases. They work in different fundamental areas of phytopathology, like detection and diagnosis, plant–pathogen interactions, disease control, disease resistance, integrated management, epidemiology, and emerging diseases, as well as more applied agronomic but also basic molecular, cellular, and biochemical approaches. Numerous advances have been made in the molecular characterization of pathogen–host interactions by scientists in Spain in recent years.

Viruses are ubiquitous in nature and can infect all kinds of organisms, including plants. Horticultural crops suffer periodic epidemics caused by viruses, most likely as a consequence of the intensification of horticultural practices and global commerce [32]. Research applied to the control of plant viruses is therefore of critical importance in Spain, but this must be based on a solid fundamental knowledge of plant–virus interactions. Thus, in this Special Issue of the *International Journal of Molecular Sciences,* “State-of-the-Art Molecular Plant Biology Research in Spain”, we present data on the translation of virus genomes in the infected host cell [33]. Because of the importance of basic research in plant virology, we are also pioneering a new Special Issue in this journal, entitled the “Molecular characterization of plant-virus interactions” [34].

### 3.6. Environmental Sciences

The development of and response to environmental conditions are the two most studied processes in plant biology worldwide, and Spain is not an exception. This topic is closely related to the climate change scenario and the need for breeding programs and to develop crop management strategies. Implicated genes, gene product interactions, receptors, signaling, hormone actions, transcription factors, omics profiles, and general and species-specific responses are the main questions behind this research. Identifying receptors is one of the most challenging objectives in understanding plant responses to physical, chemical, and biological stimuli. Jasmonic acid, gibberellins, auxins, ABA, brassinosteroids, salicylic acid, signaling, the interplay between different routes, the modulation of developmental processes, and responses to environmental conditions have been partially characterized [35]. The role of ROS and NO species, cross-talks with other plant regulators, and antioxidant chemical and enzymatic systems have been characterized [36]. Highly cited papers dealing with mineral nutritional deficiencies have been (co)-authored by Spanish scientists [37]. Here, we would like to show our recognition and gratitude towards Prof. Abadia, who recently passed away. 

**Figure 5:**
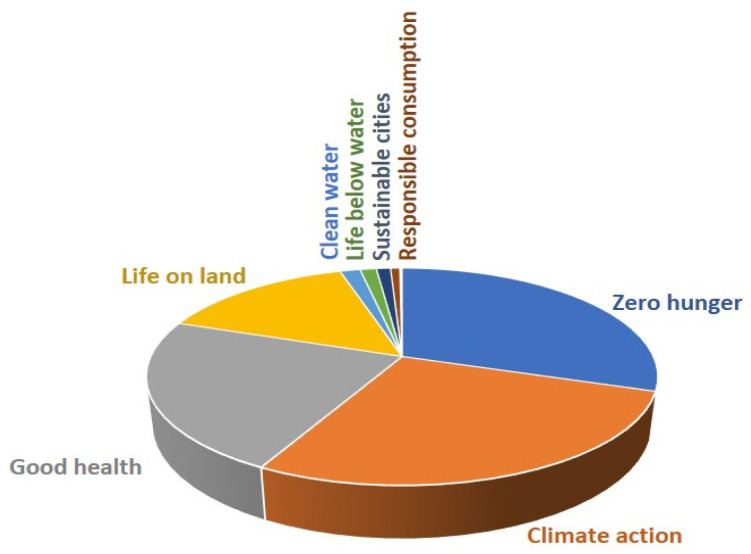
Percentage of manuscripts from Spanish groups included in the Web of Science [18] in relation to sustainable development goals.

In this context, regarding sustainable development goals, the main purpose of Spanish research has been the pursuit of zero hunger (30% of manuscripts from Spanish groups included in in the Web of Science [18]), climate action (28% of manuscripts), good health (22%), and life on land (14%), but also clean water (2%), life below water (1.5%), sustainable cities (1.5%), and responsible consumption (1%) (Figure 5).

## 4. Conclusions

To conclude, we can asseverate that molecular plant biology research is vital in Spain within the global context. In the period from 2000 to 2023, 73,263 documents were published by Spanish teams in the field of plant molecular biology in the scientific database Scopus, with 11,560 manuscripts in the more restrictive Web of Science. In this regard, Spain occupies the eighth position after the USA, China, India, Germany, the United Kingdom, Japan, and France. During these 23 years, the number of publications has increased continuously, showing Spain’s strong international position. The most important research areas include plant physiology and biochemistry, applied microbiology, genetics and heredity, cell and evolutionary biology, phytopathology, and environmental sciences. In addition, regarding sustainable development goals, the main purpose of Spanish investigations and scientific publications have been the pursuit of zero hunger, climate action, good health, and life on land.

## Data Availability

Data are contained within the article.
